# Association of *TRPV5*, *CASR*, and *CALCR* genetic variants with kidney stone disease susceptibility in Egyptians through main effects and gene–gene interactions

**DOI:** 10.1007/s00240-022-01360-z

**Published:** 2022-09-11

**Authors:** Fahmy T. Ali, Eman M. Abd El-Azeem, Hala F. A. Hekal, Mayada M. El-Gizawy, Mohamed S. Sayed, AbdAllah Y. Mandoh, Ahmed F. Soliman

**Affiliations:** 1 Biochemistry Department, Faculty of Science, Ain Shams University, Cairo, Egypt; 2 Medical Physiology Department, Medical Research Institute, National Research Centre, Giza, Egypt; 3 Urology Department, Faculty of Medicine, Ain Shams University, Cairo, Egypt; 4 Molecular Biology and Cytogenetics Department, Armed Forces Laboratories of Medical Research and Blood Bank, Cairo, Egypt

**Keywords:** Kidney stone disease (KSD), Transient receptor potential vanilloid member 5 (*TRPV5*), Calcium-sensing receptor (*CASR*), Calcitonin receptor (*CALCR*), Single nucleotide polymorphism (SNP)

## Abstract

Kidney stone disease (KSD) represents an urgent medical problem because of increasing its prevalence. Several functional polymorphisms in genes involved in the renal handling of calcium were associated with KSD pathogenesis. Among those, the rs4236480 of transient receptor potential vanilloid member 5 (*TRPV5*) gene, the rs1801725 of calcium-sensing receptor (*CASR*) gene, and the rs1801197 of calcitonin receptor (*CALCR*) gene appear to be of great importance. Due to the scarce data on the Egyptians, this study aimed to evaluate the association of these candidate genetic variants with the risk of developing KSD in an Egyptian population. To do so, the biochemical parameters were measured along with the genotyping of the three polymorphisms using allelic discrimination assay in 134 KSD patients and 86 age and sex-matched healthy subjects. The results showed that the genotypic distributions and allelic frequencies of the studied variants were significantly different between cases and controls. The three polymorphisms increased the risk of KSD significantly under all the tested genetic models (OR ranges from 2.152 to 5.994), except for the recessive model of the *CALCR* rs1801197 polymorphism after Bonferroni correction. The gene–gene interaction analyzed by multifactor dimensionality reduction selected the three-locus combination as the best model associated with the susceptibility to KSD with OR 9.706. Further, synergistic interactions were identified between *TRPV5* rs4236480 and *CALCR* rs1801197 variants and *CASR* rs1801725 and *CALCR* rs1801197 variants. In conclusion, the *TRPV5* rs4236480, *CASR* rs1801725, and *CALCR* rs1801197 polymorphisms showed a significant association with the risk of KSD in the Egyptian population. Furthermore, their complex interactions might have an impact on the genetic susceptibility to develop KSD.

## Introduction

Kidney stone disease (KSD), also known as urolithiasis, is the third most common urological disease [1]. It results from successive physicochemical events of supersaturation, nucleation, aggregation, and finally retention of crystal-forming substances in any part of the urinary system including kidney, ureter, or urinary bladder [2]. In 80% of cases, high urinary calcium concentration is responsible for stones formation in form of calcium oxalates, calcium phosphates, or a combination of oxalate and phosphate with uric acid [3]. KSD patients suffer from intense pain, hematuria, and sometimes renal failure. This makes the disorder, together with its high prevalence rate and tendency to relapse, an actual issue of modern urology [4].

KSD is a multi-factorial disorder resulting from the combined influence of epidemiological, nutritional, socio-economic, biochemical, and genetic risk factors [5]. The fact that individuals with a family history of KSD are more susceptible to kidney stone formation than the general population reflects the importance of genetic factors in KSD development [6]. As mentioned, calcium-rich stones constitute the majority of kidney stones therefore genes that regulate calcium homeostasis may represent suitable candidates for recognizing subjects at a higher risk for KSD [7].

The transient receptor potential vanilloid member 5 (*TRPV5*) gene is mapped to chromosome 7q35 and encodes for the TRPV5 trans-epithelial channel. This channel is 729 amino acids long and is expressed in renal distal convoluted tubules and collecting ducts, the major parts of the kidneys involved in calcium homeostasis, where it constitutes the rate-limiting step in calcium reabsorption [8, 9]. In mice, *TRPV5* knockdown altered renal calcium handling which led to diminished active calcium reabsorption and thus produced severe hypercalciuria; the latter is a major risk factor for kidney stone formation [10]. Moreover, significant associations were reported between the non-synonymous single nucleotide polymorphism (SNP) rs4236480 of the *TRPV5* gene and the risk of KSD in a West Bengal population of India and with kidney stone multiplicity in Taiwanese patients [11, 12].

Another gene involved in calcium homeostasis is the calcium-sensing receptor (*CASR*) gene located on chromosome 3q13.3-21. It encodes for CaSR protein which contains 1078 amino acids, belongs to the G-protein coupled receptors superfamily, and is expressed predominantly on the plasma membrane of the parathyroid gland and renal tubular cells [13]. When calcium binds to CaSR, a cascade of events occurs that eventually inhibits the secretion of parathyroid hormone (PTH) and the reabsorption of calcium in the thick ascending limb of the loop of Henle. Thus, the activation of CaSR increases the urinary excretion of calcium by both direct and PTH-mediated effects on renal tubular cells [14, 15]. Previous reports described that several SNPs in *CASR* were associated with KSD and the SNP rs1801725 appears to be the most frequent [16, 17].

The calcitonin receptor (CALCR) is another seven-transmembrane G-protein coupled receptor located on the epithelial cells of the thick ascending limb of the loop of Henle, distal convoluted tubules, and collecting ducts. It is encoded by the *CALCR* gene located on chromosome 7q21.3 [18]. Calcitonin, a calcium metabolism-related hormone, increases absolute calcium reabsorption and decreases phosphate reabsorption in renal tubules by binding to CALCR [19]. Therefore, *CALCR* has been proposed as a candidate genetic marker for the predisposition to KSD. Several polymorphic sites, mostly SNPs, were found in the *CALCR* gene. Among them, the variant rs1801197 was associated with KSD [20–22].

Hence and because there are no earlier reports in Egypt, the current study was attempted to analyze *TRPV5* rs4236480, *CASR* rs1801725, and *CALCR* rs1801197 gene polymorphisms to evaluate their association with KSD in an Egyptian population.

## Subjects and methods

### Study population

This is a case–control study that sequentially recruited 134 radiologically proven KSD patients with calcium-rich stone**s** (calcium oxalate and/or calcium phosphate) or with a history of surgical removal of such stones from the Department of Urology, El-Demerdash Hospital, Ain Shams University**,** in the period between July 2018 and February 2021. KSD was documented by a plain X-ray film and renal ultrasound while the stone composition was verified using X-ray crystallography. Patients suffering from metabolic, gastrointestinal, renal, or endocrine disorders in addition to patients taking drugs such steroids, diuretics, or those affecting electrolyte or citrate handling (vitamin D, etc.) were excluded. Additionally, a control group comprised 86 age- and sex-matched subjects who had no familial KSD history and renal calcification as evident from the renal ultrasonography.

Written informed consent was received from all participants before the start of the study. This work is conformed to The Declaration of Helsinki and approved by the local Ethics Committee of Faculty of Medicine, Ain Shams University, Egypt.

From each participant, a volume of 3 ml of venous blood was collected into dry tubes, left to clot, and centrifuged to obtain sera for biochemical analyses. In addition, 2 ml were collected into ethylene diamine tetraacetic acid (EDTA) coated tubes for molecular analyses. Moreover, a 24 h urinary excretion was collected from all participants.

### Laboratory analyses

Blood urea nitrogen (BUN), creatinine, total calcium, and phosphorus levels were determined in sera of all participants alongside the urinary calcium and phosphorus levels using Siemens Dimension RxL Max Integrated Chemistry System (Siemens Healthcare Diagnostics, DE, USA).

### Genotyping

The *TRPV5* rs4236480, the *CASR* rs1801725, and the *CALCR* rs1801197 gene polymorphisms were genotyped using allelic discrimination (AD) assay. Briefly, genomic DNA was extracted from whole blood samples using QIAamp^®^ DNA blood mini kit (Qiagen, Hilden, Germany). Real-time PCR reactions were performed in a final volume of 20 μl with ~ 20 ng of DNA. Exactly 0.5 μl of 20 × working stock solution of the SNP genotyping assay containing the primers and probes for the gene of interest as part of the kit (Cat# 4351379, assay ID: C_67881_10 for *TRPV5*, C_7504853_20 for *CASR* and C_2541576_1 for *CALCR*; respectively) (Applied Biosystems) was added to 10 μl of 2 × TaqMan^®^ universal PCR master mix (Applied Biosystems) then the reaction’s volume was completed with water. PCR conditions were as follows: 10 min at 95 °C for AmpliTaq Gold enzyme activation and initial denaturation followed by 40 amplification cycles of denaturation at 92 °C for 15 s and annealing/extension at 60 °C for 60 s. PCR reactions were carried out in MicroAmp^®^ fast optical 96-Well reaction plate with MicroAmp^®^ optical adhesive film (Applied Biosystems) The plate was loaded into the 7500 Real-Time PCR System (Applied Biosystems, CA, USA) and the fluorescence data were analyzed in genotyping mode by the instrument's software.

### Statistical analysis

The assumption of Gaussian distribution was tested with the Shapiro–Wilk test; the Gaussian distributed data were expressed as mean ± SD, non-Gaussian distributed data were expressed as median and inter-quartile range (25th and 75th percentile), and categorical variables are expressed as frequencies (percentages). Continuous variables were compared between the two groups using Student’s *t* test, or Mann–Whitney *U* test as appropriate. *χ*^2^ test was used to compare the differences between categorical variables and to assess the departures from Hardy–Weinberg equilibrium (HWE). The association between the studied polymorphisms and the susceptibility to KSD was investigated under various genetic models using unconditional logistic regression analyses; adjusted odd ratio (OR) for sex and age and their corresponding 95% confidence interval (CI) were used to measure the strength of association. All *p*-values were 2-sided, and a *p*-value < 0.05 was considered statistically significant. For association analyses, a statistical significance threshold was set to *p* < 0.0167 after Bonferroni correction. Statistical analyses were performed using SPSS version 20 (IBM Corp, NY, USA). Power calculations of sample size were carried out using the Bioinformatics Institute’s Online Sample Size Estimator (OSSE).

For detecting multi-loci genotype combinations which may predict KSD risk, the multifactor dimensionality reduction (MDR) approach was applied using the MDR software version 3.0.2 (Computational Genetics Laboratory, Institute for Quantitative Biomedical Sciences, NH, USA). The best model for defining KSD risk was found using cross-validation consistency (CVC) and testing balanced accuracy (TBA). CVC is defined as the number of times that a particular SNP-SNP combination is identified out of ten cross-validations. To compare the observed testing balanced accuracy with that expected under the null hypothesis of no association, the statistical significance was further evaluated after a 1000-fold permutation testing using the MDR Permutation Testing Module version 1.0 beta 2.

## Results

### Basic characteristics of the study population

General characteristics of the study population are shown in Table 1. The two groups did not show significant differences in age and sex, in addition to the serum creatinine, calcium, and phosphorus levels alongside the urinary phosphorus concentration. Compared to controls, KSD patients had significantly higher BUN and urinary calcium levels.

**Table 1:** General characteristics of the study subjects

	Control group (*n* = 86)	KSD group (*n* = 134)	*p*-value
Age (years)	42.02 ± 9.06	42.00 (35.00–50.00)	0.627
Gender (M/F)	46 (53.5)/40 (46.5)	78 (58.2)/56 (41.8)	0.491
BUN (mg/dl)	12.95 ± 3.25	17.00 (13.00–22.00)	< 0.001
Serum creatinine (mg/dl)	0.92 ± 0.16	0.90 (0.80–1.10)	0.174
Serum total calcium (mg/dl)	9.10 (8.80–9.30)	9.20 ± 0.52	0.372
Serum phosphorus (mg/dl)	3.84 ± 0.53	3.80 (3.40–4.10)	0.134
Urinary calcium (mg/24 h)	172.00 (146.00–198.00)	201.00 (190.00–220.00)	< 0.001
Urinary phosphorus (mg/24 h)	547.00 (493.00–612.00)	572.00 (49.00–688.00)	0.126

Data are expressed as mean ± SD for Gaussian variables, median (inter-quartile range) for non-Gaussian variables, and frequencies (percentages) for categorical variables

*KSD* Kidney stone disease, *BUN* blood urea nitrogen

### Genotypic distribution and allelic frequencies

Table 2 illustrates some details of the studied SNPs, whereas Table 3 shows the genotypic distribution and allelic frequencies of the studied polymorphisms between controls and KSD patients. The genotypic distribution of the three polymorphisms fitted HWE in both groups (*p* > 0.05). For *TRPV5* rs4236480 SNP, KSD patients showed a significantly higher frequency of the homozygous mutant (TT) genotype with a decrease in the homozygous wild (CC) genotype compared to controls (*p* = 0.001). Hence, KSD patients had a higher frequency of the minor T allele than normal subjects (*p* < 0.001). The same results were observed when considering *CASR* rs1801725 SNP with a significantly higher frequency of TT genotype and a decrease in GG genotype in KSD patients compared to the control group (*p* < 0.001). Accordingly, the frequency of the mutant T allele carriers in KSD patients was higher than that in normal subjects (*p* < 0.001).

**Table 2:** Some details of the variants under study

Gene	dbSNP	Nucleotide variation	Amino acid variation	Global MAF (%)
*TRPV5*	rs4236480	c.461A>G	p.H154R	*T* = 32.7 (ALFA project) *T* = 40.5 (HapMap)
*CASR*	rs1801725	c.2473G>T	p.A986S	*T* = 14.3 (ALFA project) *T* = 7.3 (HapMap) *T* = 9.4 (1000 Genomes project)
*CALCR*	rs1801197	c.1340T>C	p.L447P	*G* = 29.7 (ALFA project) *A* = 42.6 (HapMap) *A* = 45.6 (1000 Genomes project)

**Table 3:** Genotype distribution and allele frequencies of the studied genes among KSD patients compared to control subjects

SNP	Group	Genotype distribution *n* (%)			*p*-HWE	*p*-value	Allele frequency (%)		*p*-value
*TRPV5* rs4236480		CC	CT	TT			C	T	
	Control	40 (46.51)	32 (37.21)	14 (16.28)	0.070	0.001	65.12	34.88	< 0.001
	KSD	32 (23.88)	56 (41.79)	46 (34.33)	0.121		44.78	55.22	
*CASR* rs1801725		GG	GT	TT			G	T	
	Control	44 (51.16)	36 (41.86)	6 (6.98)	0.690	< 0.001	72.09	27.91	< 0.001
	KSD	38 (28.36)	66 (49.25)	30 (22.39)	0.909		52.99	47.01	
*CALCR* rs1801197		GG	GA	AA			G	A	
	Control	44 (51.16)	32 (37.21)	10 (11.6)	0.239	0.002	69.77	30.23	< 0.001
	KSD	38 (28.36)	64 (47.76)	32 (23.88)	0.667		52.24	47.76	

Data are expressed as frequencies (percentage)

*KSD* Kidney stone disease, *SNP* single nucleotide polymorphism, *TRPV5* transient receptor potential vanilloid member 5, *CASR* calcium-sensing receptor, *CALCR* calcitonin receptor, *p*-*HWE* *p*-value of Hardy–Weinberg equilibrium

Referring to *CALCR* rs1801197, KSD patients showed a significantly higher frequency of AA and GA genotypes with a decrease in GG genotype compared to controls (*p* = 0.002). Therefore, KSD patients had a higher frequency of the polymorphic A allele than normal subjects (*p* < 0.001).

The power calculations showed that the sample size of the present study can give, at the level of *α* error probability = 0.05, as high as 83.4, 81.5 and 76.5% power for the *TRPV5*, *CASR*, and *CALCR* gene polymorphisms; respectively. Accordingly, our sample size can give sufficient power to accept/deny the association, except for the *CALCR* gene polymorphism.

### Gene polymorphisms and KSD risk

Codominant, dominant, recessive, and allelic genetic models were applied to test the associations of the *TRPV5*, *CASR*, and *CALCR* polymorphisms with the risk of KSD development (Table 4). The *TRPV5* rs4236480 SNP was associated with increased susceptibility to develop KSD under all the tested genetic models (*p* < 0.001, *p* = 0.011, *p* < 0.001, *p* = 0.004, and *p* < 0.001; respectively).

**Table 4:** Association of *TRPV5* and *CASR* variants with KSD risk according to the genetic association models

	Allelic model	Homozygous codominant model	Heterozygous codominant model	Dominant model	Recessive model
*TRPV5* rs4236480	T vs C	TT vs CC	CT vs CC	TT/CT vs CC	TT vs CT/CC
^a^Adjusted OR (95% CI)	2.347 (1.575–3.499)	4.317 (1.975–9.443)	2.314 (1.210–4.428)	2.886 (1.602–5.200)	2.760 (1.389–5.483)
*p*-value	< 0.001	< 0.001	0.011	< 0.001	0.004
*CASR* rs1801725	T vs G	TT vs GG	GT vs GG	TT/GT vs GG	TT vs GT/GG
^a^Adjusted OR (95% CI)	2.325 (1.539–3.511)	5.994 (2.232–16.094)	2.209 (1.199–4.068)	2.839 (1.589–5.071)	3.790 (1.501–9.569)
*p*-value	< 0.001	< 0.001	0.011	< 0.001	0.005
*CALCR* rs1801197	A vs G	AA vs GG	GA vs GG	AA/GA vs GG	AA vs GA/GG
^a^Adjusted OR (95% CI)	2.152 (1.434–3.232)	4.102 (1.746–9.637)	2.746 (1.435–5.255)	2.899 (1.616–5.215)	2.378 (1.095–5.161)
*p*-value	< 0.001	0.001	0.002	< 0.001	0.029

*KSD* Kidney stone disease, *TRPV5* transient receptor potential vanilloid member 5, *CASR* calcium-sensing receptor, *CALCR* calcitonin receptor, *OR* odd ratio, 95% *CI* 95% confidence interval

^a^ Adjusted for sex and age

Regarding *CASR* rs1801725 SNP, the polymorphism was associated with high KSD risk under all the tested genetic models (*p* < 0.001, *p* = 0.011, *p* < 0.001, *p* = 0.005, and *p* < 0.001; respectively).

On the other hand, the *CALCR* rs1801197 SNP was associated with an increased risk of KSD under the homozygous codominant, heterozygous codominant, dominant, and allelic models only (*p* = 0.001, *p* = 0.002, *p* < 0.001, and *p* < 0.001; respectively). Whereas, the association under the recessive model did not survive after the correction for multiple hypotheses (*p* = 0.029).

### Gene–gene interaction analysis

**Fig. 1:**
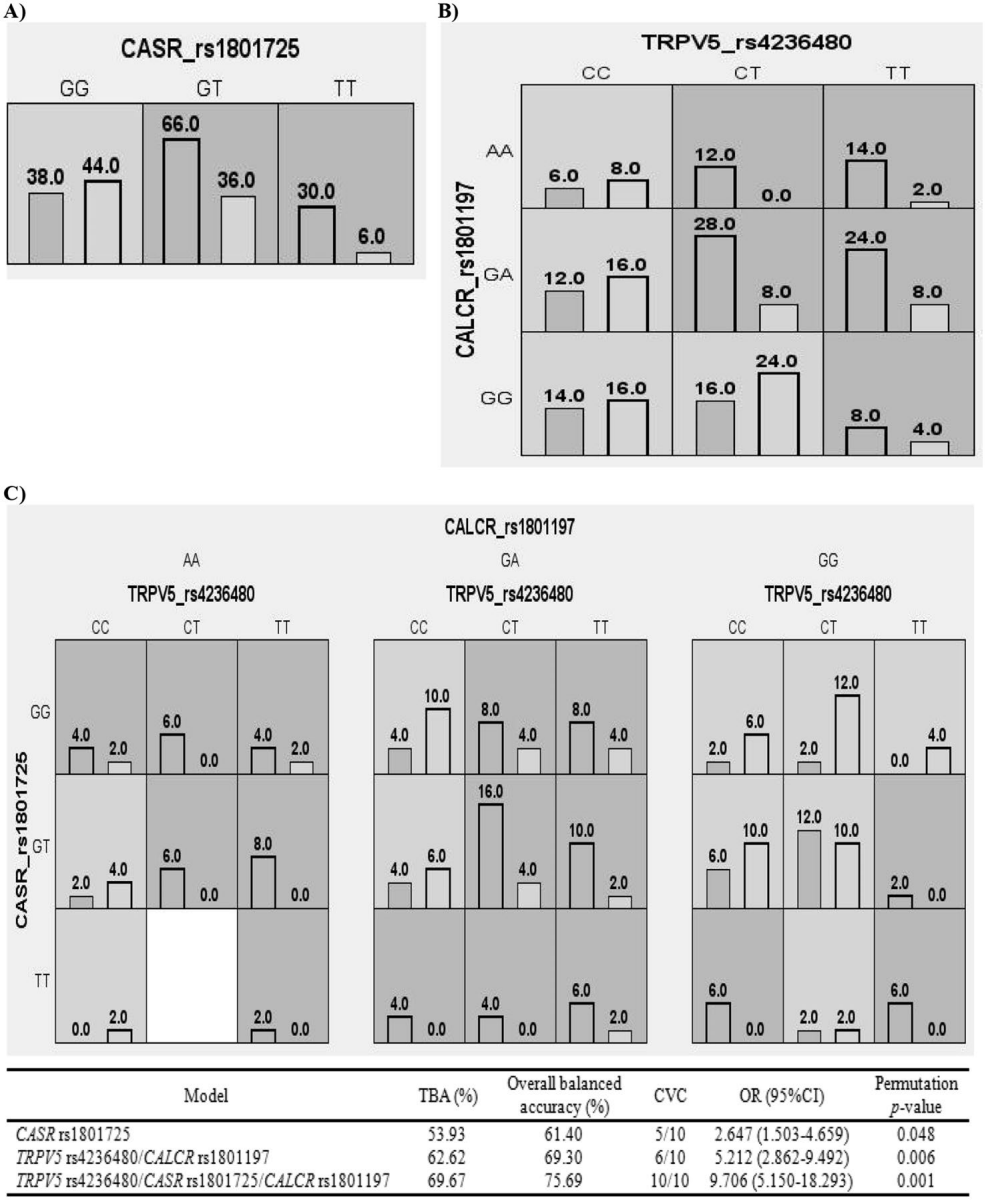
Distribution of high-risk and low-risk genotypes in the optimal models as detected by multifactor dimensionality reduction (MDR) analysis. **A** The best one-factor model, **B** the best two-way model, and **C** the best multi-loci model to predict the risk of KSD. The dark and light shading boxes represent the high-risk and low-risk combinations, respectively. For each model, the left and right bars in the boxes represent the number of KSD patients and control subjects, respectively. Boxes were labeled as high-risk if the ratio of the number of cases to controls met or exceeded the threshold of 1.558. Based on the pattern of high-risk and low-risk genotypes, the two- and three-locus models are evidence of gene–gene interaction. *TRPV5* Transient receptor potential vanilloid member 5, *CASR* calcium-sensing receptor, *CALCR* calcitonin receptor, *TBA* testing balanced accuracy, *CVC* cross-validation consistency, *OR* odd ratio, *CI* confidence interval

Figure 1 summarizes the results of the exhaustive MDR analysis evaluating all possible combinations of the studied polymorphisms. Among the three loci, *CASR* rs1801725 seems to be the best one-factor model to predict the risk of KSD while the best two-way model was the combination of *TRPV5* rs4236480 and *CALCR* rs1801197 loci. According to the MDR analysis, the best model predicting a potential KSD risk was that of the three-locus interaction consisting of *TRPV5* rs4236480, *CASR* rs1801725, and *CALCR* rs1801197 which showed the highest TBA of 69.67% and CVC of 10/10 with OR 9.706, 95% CI 5.150–18.293, and a *p*-value of the 1000-fold permutation testing = 0.001.

**Fig. 2:**
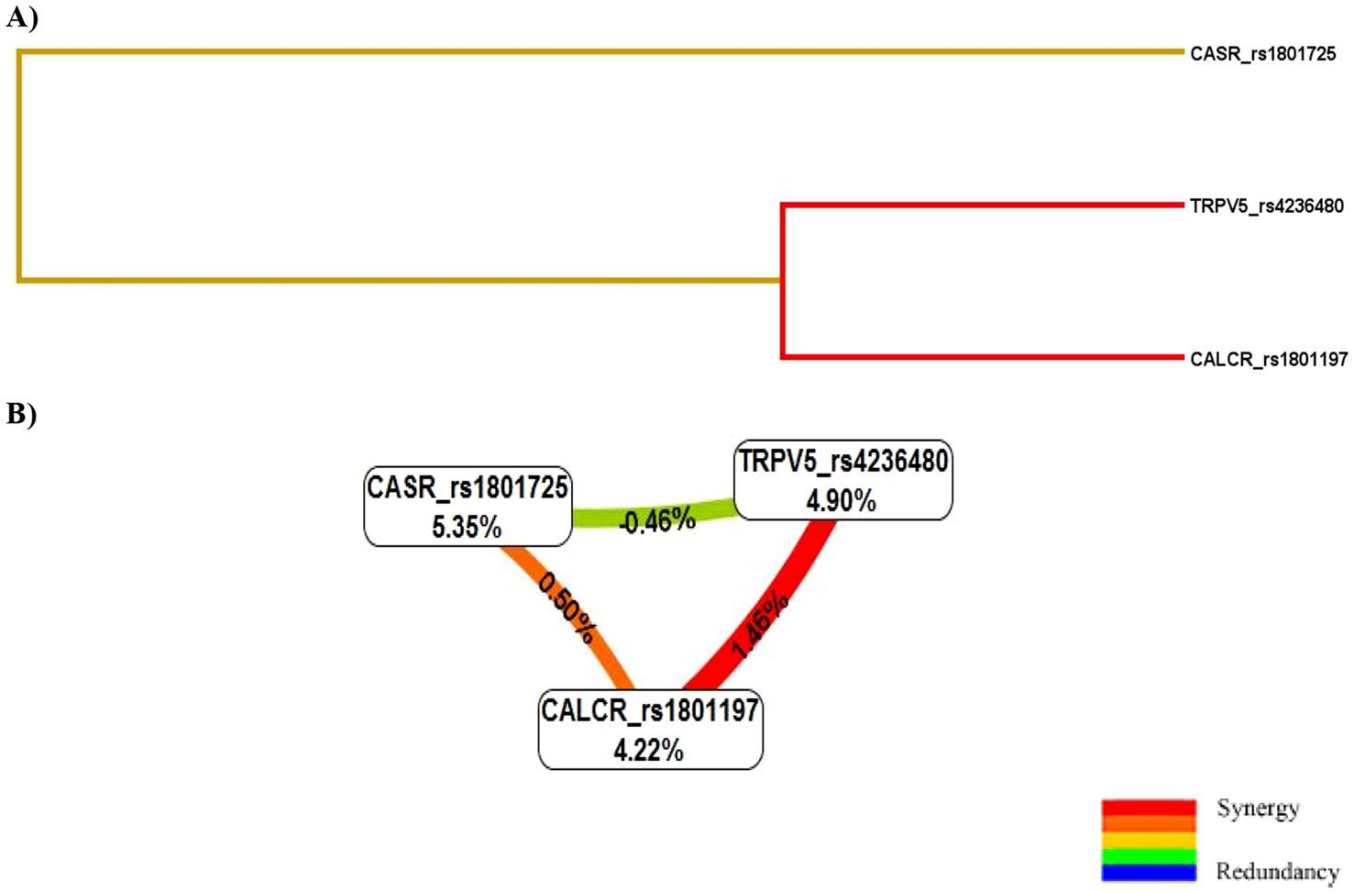
KSD-related genetic interaction networks among *TRPV5*, *CASR*, and *CALCR* derived from multifactor dimensionality reduction (MDR). **A** Dendrogram of the interaction between the three polymorphisms in response to the presence of KSD. The strength of the interactions is shown by the distance between the genes; the short connection represents a stronger synergistic reaction. **B** Fruchterman-Reingold graph describes the percentage of the entropy (information gain) that is explained by each factor or 2-way interaction. Values inside nodes indicate information gain of individual attributes or main effects, whereas values between nodes show information gain of pairwise combinations of attributes or interaction effects. Positive entropy plotted in red or orange indicates a synergistic interaction while negative entropy plotted in green indicates independence or redundancy. *TRPV5* Transient receptor potential vanilloid member 5, *CASR* calcium-sensing receptor, *CALCR* calcitonin receptor

Figure 2 shows dendrogram and Fruchterman-Reingold graph that describe the interactions between the three SNPs. The patterns of entropy recapitulate the main and/or interaction effect for each pairwise combination of attributes. The strongest interaction effect was found between *TRPV5* rs4236480 and *CALCR* rs1801197 with the information gain (IG) value of 1.46%, suggesting that the two polymorphisms have a synergistic interaction sharing the positive IG concerning KSD. Further, a more weakly synergistic effect on KSD risk was found between *CASR* rs1801725 and *CALCR* rs1801197 with an IG value of 0.50%.

## Discussion

KSD is a costly disease that imposes huge cost implications on the patient and healthcare systems. Because the disease results, in most cases, from calcium concrements formation, there is a ground for research into calcium metabolism impairments in KSD patients. Further, interactions between multiple environmental and genetic factors are believed to be involved in the pathogenesis of KSD [4, 23]. Thus, association studies have attempted to assess the role of candidate genes in the development of KSD [24]. Consequently, this work was conducted to evaluate the association of *TRPV5* rs4236480, *CASR* rs1801725, and *CALCR* rs1801197 gene polymorphisms with the risk of KSD in an Egyptian population.

In the present study, no deviation from HWE was found in the genotype distribution of the SNPs under study. On the other hand, there was no enough data about the allele frequencies of the studied polymorphisms, to the best of our knowledge, in the Egyptian population. However, the C allele of the *TRPV5* rs4236480, the G allele of the *CASR* rs1801725 and the G allele of the *CALCR* rs1801197 SNPs were the most prominent in controls of the current work in agreement with the results observed in previous studies [11, 16, 20, 21, 25, 26].

TRPV5 is a tetramer channel protein consisting of six transmembrane helices, an N-terminal ankyrin repeats domain (ARD), and a TRP domain [27]. The *TRPV5* rs4236480 polymorphism causes a substitution of arginine for histidine at position 154 located in the third finger loop of the N-terminal ARD. This domain forms a binding interface for the neighboring subunits, thus, it was suggested to play an important role in channel tetramerization [28, 29]. Therefore, mutations in the interacting residues could decrease the stability of the ARD, thereby affecting the overall fold and preventing channel assembly.

There are limited clinical studies with controversial results about the association of *TRPV5* rs4236480 polymorphism with KSD. Mitra et al. [11] and Khaleel et al. [12] found significant associations between the presence of the rs4236480 variant and increased risk of kidney stone formation and kidney stone multiplicity, respectively. This is in line with the results of the present work which showed that the occurrence of KSD was higher in individuals carrying one minor allele, at least, of the *TRPV5* rs4236480 polymorphism in comparison with the carriers of the major allele. On the other hand, Na et al. [30] and Renkema et al. [31] demonstrated a non-significant impact of the *TRPV5* rs4236480 SNP. TRPV5 plays a crucial role in regulating urinary calcium levels by mediating the transport and reabsorption of calcium in the kidney [10, 30]. Previously, the expression levels of TRPV5 protein and mRNA were found to be significantly decreased in genetic hypercalciuric stone-forming rats compared to normal controls [32]. Moreover, the expression level of TPRV5 protein was lower in the stone-affected region compared to the adjacent control tissues in KSD patients [11]. TRPV5 down-regulation could decrease calcium reabsorption and exaggerate its urinary excretion leading to hypercalciuria which per se is a recognizable risk factor in stone formation [32].

Regarding CaSR, it is composed of extracellular, heptahelical transmembrane, and intracellular C-terminal domains [33]. The *CASR* rs1801725 SNP causes a substitution of an alanine amino acid with a serine residue at position 986 residing in the cytoplasmic tail of CaSR. Mutations within the respective region, according to the in vitro studies, may influence CaSR function and impair signal transduction, intracellular trafficking, and/or cell surface expression [34–36]. Moreover, Wang et al. [37] suggested that the variant allele of the *CASR* rs1801725 polymorphism may result in the production of a less active receptor.

Although several reports studied the association of the *CASR* rs1801725 polymorphism with the risk of KSD, the results were conflicting. Some studies detected an association with an increased risk to develop KSD [16, 17] whereas others did not confer a significant association [25, 26, 38, 39]. In congruence with the positive findings, the results of the current work revealed that the TT and GT carriers of the *CASR* rs1801725 SNP had an increased risk to develop KSD compared to their corresponding wild-type carriers. In the kidney, CaSR prevents the reabsorption of divalent cations in the thick ascending limb of the loop of Henle, triggers the inhibitory actions of hypercalcemia on the urinary-concentrating mechanism, and subsequently prevents kidney stone formation [40, 41]. Reduced CaSR expression may prompt stones formation in the renal medulla due to alteration of the normal balance among calcium, phosphate, protons, and water excretion causing calcium phosphate crystals intratubular precipitation and consequently calcium phosphate stone formation [42].

Additionally, it increases the predisposition of calcium to precipitate in the papillary interstitium and the possible consequent formation of Randall’s plaque on which calcium oxalate stones develop [43].

When considering CALCR, the rs1801197 polymorphism alters the encoded amino acid from proline located in the third intracellular C-terminal domain of the receptor at position 447 to leucine [21, 44]. This change could alter the secondary structure of the receptor by changing its net charge, and thus affects the affinity for ligand binding, the G-protein signal transduction, and thus the reaction of the target cells toward calcitonin [45, 46].

The association between the *CALCR* rs1801197 polymorphism and the development of KSD was the focus of a few studies, and the results were inconsistent. On one hand, a significant association was reported in different ethnic populations [20, 21, 46, 47]. Moreover, a meta-analysis performed by Qin et al. [48] confirmed the association between the *CALCR* rs1801197 polymorphism and KSD pathogenesis. This is in agreement with the results of the present study revealing that the individuals with the mutant A allele were more prone to KSD than those with the wild G allele. On the other hand, Mittal et al. [22] and Shakhssalim et al. [49] reported a non-significant association.

The variability between the results of the current work and other studies can be attributed to the complexity of the disease etiology, genetic heterogeneity of the disease, ethnicity, and the differences in the study population characteristics including, but not limited to, social life patterns and habits, gene–gene and gene-environment interactions, sample size, and selection of the control group.

In a polygenic disease such as KSD, studying the gene–gene interactions and investigating the complex impact of gene polymorphisms, where the effect of the variation of a single gene is influenced by other genetic variations, are no less important for determining the risk of the disease development. In this sense, the MDR used to analyze the interactions of the three SNPs revealed that the *CASR* rs1801725 was the best one-factor model and that the two-locus model consisting of *TRPV5* rs4236480 and *CALCR* rs1801197 conferred OR of 5.212. Meanwhile, the best multi-loci model was a combination of the three polymorphisms. Moreover, the interaction dendrogram and Fruchterman-Reingold graph indicated a synergistic interaction between loci rs4236480 of the *TRPV5* gene and rs1801197 of the *CALCR* gene that contributed to increased susceptibility to KSD. This highlights the importance of epistasis testing, illustrates the complexity of the disease, and indicates that the susceptibility may be modulated not only by a variety of genetic factors but also by non-linear gene–gene interactions.

In conclusion, the present study is the first, to the best of our knowledge, in Egypt and North Africa that address the association between *TRPV5*, *CASR*, and *CALCR* gene polymorphisms with KSD. The results showed a significant association between *TRPV5* rs4236480, *CASR* rs1801725, and *CALCR* rs1801197 SNPs and the risk of developing KSD. Further, the effect of interactions between these polymorphisms might have an impact on genetic susceptibility to develop KSD. Consequently, these genes appear to be suitable candidates to explain the individual predisposition to KSD and might help for a better diagnosis of this complex disease.

### Study limitation

Several limitations should be acknowledged in this study. First, one of the three SNPs under study, *CALCR* rs1801197, may lack sufficient power to deny/accept the association. Second, the effect of potential gene-environmental interactions was not identified because of the lack of information about environmental factors such as diet and behavior (e.g., alcohol drinking). Third, this was a study with a modest sample size and limited SNPs in calcium homeostasis-related genes. Finally, there are likely selection bias and limitation of the generalizability of the findings because this was a single-center study; for that, multicenter studies with a larger sample size are needed to confirm the results of the present work.

## Data Availability

The datasets used and/or analyzed during the current study are available from the corresponding author on reasonable request.
